# Sex specific serum uric acid levels are associated with ischemic changes on ECG and with 20-year all-cause mortality among older adults

**DOI:** 10.1371/journal.pone.0283839

**Published:** 2023-03-30

**Authors:** Yonatan Moshkovits, Adam Goldman, Angela Chetrit, Rachel Dankner

**Affiliations:** 1 Leviev Heart Center, Sheba Medical Center, Ramat Gan, Israel; 2 Unit for Cardiovascular Epidemiology, The Gertner Institute for Epidemiology and Health Policy Research, Sheba Medical Center, Ramat Gan, Israel; 3 Department of Epidemiology and Preventive Medicine, School of Public Health, Sackler School of Medicine, Tel Aviv University, Tel Aviv-Yafo, Israel; Bolu Abant İzzet Baysal University: Bolu Abant Izzet Baysal Universitesi, TURKEY

## Abstract

**Background:**

Uric acid is an emerging biomarker for cardiovascular morbidity and mortality, but its association with all-cause mortality and ECG findings remains unestablished, specifically among older adults. We aimed to evaluate the association between serum uric acid (SUA) with incidental findings of ECG abnormalities and with long-term all-cause mortality.

**Methods:**

We conducted a prospective cohort study of 851 community dwelling men and women, who were examined between 1999 and 2008, and followed over 20 years until December 2019 for all-cause mortality. Subjects free of Gout or diuretics treatment at baseline were included. SUA was categorized according to sex-specific tertiles and evaluated against baseline ECG findings and all-cause mortality.

**Results:**

Mean baseline age was 72±7 years and 416 (49%) were females. Ischemic changes on ECG were observed in 85 (10.0%) participants, of them 36 (13.5%) belonged to the upper SUA tertile and 49 (8.4%) to the lower ones (p = 0.02). Multivariable logistic regression showed 80% higher odds for ischemic changes on ECG among participants in the high SUA tertile (adjusted-OR = 1.8, 95%CI 1.1–2.9, p = 0.03) compared with the lower SUA two-tertiles. During a median follow-up of 14 years, 380 (44.7%) participants died. SUA ≥5.3 mg/dl for women and ≥ 6.2 mg/dl for men, was associated with a 30% greater risk for all-cause mortality in a multivariable Cox regression model (HR = 1.3, 95%CI: 1.0–1.6, p = 0.03).

**Conclusions:**

High SUA level was associated with ischemic changes on ECG and with an increased risk for all-cause mortality over 20 years of follow-up among community dwelling older adults free of Gout. Even lower sex-specific thresholds of SUA were associated with all-cause mortality than previously proposed. SUA should be considered as a biomarker for cardiovascular risk and all-cause mortality.

## Introduction

Cardiovascular (CV) mortality remains the most common cause of death today with an estimated 17.9 million deaths worldwide each year [1]. While death rates are in decline in the western world [2], CV morbidity imposes a major burden on health systems worldwide [1]. In order to reduce morbidity and mortality, CV risk factors are constantly updated and targeted.

Uric acid is the end-product of purine metabolism, derived from the breakdown of dietary or endogenous nucleic acid [3]. Serum uric acid (SUA) levels are determined by the endogenous balance of both production and excretions that are mainly correlated with sex, age, renal function, cell turnover, dietary intake of alcohol, meat, and other purine-rich foods or medications such as diuretics [3] and with inflammatory state [4, 5]. Although SUA was hypothesized to associate with cardiovascular diseases (CVD) decades ago [6], it is currently evaluated as an innovative biomarker for cerebral vascular accident (CVA), coronary heart disease (CHD), and even heart failure [7]. Furthermore, a growing body of evidence suggests an independent association between SUA and ECG abnormalities such as left ventricular hypertrophy [8, 9], atrial fibrillation [10], and tachyarrhythmia’s [11], while the association with other abnormalities such as ischemic changes is still undetermined.

The association between SUA and all-cause and CV mortality remains inconclusive [12–14]. While several systematic reviews with meta-analyses have demonstrated an independent, yet modest association with CHD, CV and all-cause mortality [15, 16] others failed to show such an association after adjustment for CV risk factors [17] or sex [14]. In the elderly population, the association remains limited with conflicting results [18, 19]. Furthermore, due to multiple risk factors for mortality among older adults, it is unclear whether the association observed in younger individuals could be extrapolated to older adults.

We therefore aim to evaluate the association of SUA, categorized into sex-specific SUA tertiles (tertiles determined separately in females and in men) with ECG abnormalities and all-cause mortality, in a cohort of community dwelling older adults over 20 years of follow-up.

## Materials and methods

### Study population

This study consists of community dwelling older adult men and women examined as part of the third phase of the Israel study on Glucose intolerance, Obesity, and Hypertension (GOH). The GOH is a prospective cohort study that initiated in 1967 and includes 5,710 participants randomly drawn from the National Population Registry according to sex, origin (Yemenite, Asian, North Africans, and European-North Americans) and birth decade (1912–1921; 1922–1931; 1932–1941). During the third phase (1999–2008), 962 survivors underwent medical interviews, extensive blood tests (fasting glucose, creatinine, lipid profile and serum uric acid) and measurements of weight, height, and resting ECG recording at regional medical centers. Blood tests and particularly uric acid were routinely examined without any specific medical indication. Data on baseline co-morbidities, family history of IHD, physical activity, medications, and specific diets associated with SUA levels (i.e. low protein diet and vegan/vegetarian diet) were reported using self-questionnaires. Further information regarding the study population and protocols are detailed elsewhere [20–22]. Blood tests were analyzed by a single laboratory. Participants signed a written informed consent to participate in the study prior to the medical examination and blood tests. The protocol was approved by the Sheba Medical Center’s IRB.

### ECG findings

ECG recordings were manually interpreted by a single cardiologist according to the Minnesota code classification system [23] and classified into 7 groups (S1 Table): nonspecific ST-T changes, ventricular strain/ hypertrophy, atrial enlargement/disease, conduction disorder, major arrhythmias, ischemic changes and other.

### Inclusion and exclusion criteria

All cohort members had test results of SUA (n = 962). Exclusion criteria included individuals with current diuretic use (n = 107) or with the diagnosis of Gout (n = 4) which was based on self-report of morbidities and chronic medications. The final sample showed similar characteristics as the initial cohort in sex, ethnicity, baseline diabetes, and smoking. The final cohort included 851 subjects.

### Study endpoints and covariates

The first analysis was cross sectional and examined SUA against the prevalence of any ECG abnormality according to the Minnesota code classification system, recorded at baseline.

The second analysis was longitudinal with all-cause mortality as an endpoint, obtained from the National Population Registry. Follow-up was initiated at date of examination and ended at the time of death or end of follow up (December-2019), whichever came first. Specific cause of death was available until December 2016 and reported using the International Classification of Diseases (ICD) 9 or ICD 10.

SUA was classified according to sex-specific tertiles, where the upper tertile was defined as high SUA and the lower two tertiles as low SUA. Thus, low SUA was classified as SUA <5.3 mg/dl and <6.2 mg/dl for women and men, respectively while high SUA was defined as SUA ≥5.3 mg/dl and ≥ 6.2 mg/dl for women and men, respectively.

### Statistical analysis

Continuous normally distributed variables were presented as mean (±standard deviation), while non-normally distributed variables were presented by median [interquartile range (IQR)]. In-group differences of continuous variables were examined using the Student T test or the Mann-Whitney test for non-normally distributed variables. Normality distribution of variables was assessed quantitatively by the Kolmogorov -Smirnov test as well as graphically using histograms. The Chi square test or Fisher’s exact test for small cells were used to compare categorical variables. Multivariable logistic regression model was used to evaluate the association between SUA and incidental ECG findings, presented by Odd Ratios with 95% confidence interval (95%CI). The model was adjusted for age, sex, ethnicity (originating in Yemen/Aden, Asia, North Africa, and Europe/North America), current smoking, measured blood pressure, current physical activity (reported using self-questionnaires), low estimated glomerular filtration rate (eGFR, defined as eGFR < 60 (mL/min) according to the Cockcroft and Gault formula), total cholesterol, abnormal fasting glucose (defined as fasting glucose ≥ 100 mg/dl), history of myocardial infraction (MI), and SUA.

The association between SUA and all-cause mortality was assessed using the Cox proportional hazards model, adjusted for the same covariates as mentioned above and presented by Hazard Ratios with 95%CI.

Interaction analysis was performed between SUA and selected covariates by incorporating a cross-term product of SUA with the covariate in the multivariable Cox regression model.

Sample size was calculated using WINPEPI software, with 80% power and two sided 5% significance level. We assumed an average probability of 45% survival at the end of follow-up, and that 840 subjects will be required to detect a Hazard ratio of 1.3.

All analyses were performed with SPSS software (version 25) and R software version 3.4.4 (R foundation for Statistical Computing). Statistical significance was defined as a two-sided p-value of ≤ 0.05.

## Results

### Baseline characteristics

The final cohort comprised of 851 subjects who met the inclusion criteria (Table 1).

**Table 1:** Baseline characteristics of 851 community dwelling elderly men and women according to serum uric acid* upper vs. two lower tertiles^‡^.

	Low SUA n = 584	High SUA n = 267	p-value
Age (years), mean ± SD	71.8 ± 7	72.2 ± 7	0.5
Female, %	49	47	0.6
Origin, %			0.02
Middle East	29	23	
North Africa	14	22	
Yemen	23	21	
Europe-America	34	34	
Active smokers, %	11	9	0.3
Physically active, current, %	54	48	0.09
BMI, (kg/m^2^), mean ± SD	27.8±6.6	29.5±4.6	<0.001
Normal, %^a^	27	14	<0.001
Overweight, %^a^	49	43	
Obese, %^a^	24	43	
Systolic blood pressure (mmHg), mean ± SD	136.2 ± 20	138.7 ± 18	0.1
Diastolic blood pressure (mmHg), mean ± SD	77.3 ± 11	77.9 ± 10	0.2
Total cholesterol (mg/dL), mean ± SD	214.3 ± 44	215 ± 45	0.8
High total cholesterol, %^b^	61	61	0.9
Triglycerides (mg/dL), median [IQR]	112 [68]	139 [77]	<0.001
Fasting glucose (mg/dL), mean ± SD	112 ± 40	118 ± 37	0.05
Glycemic state: ^c^			<0.001
Normoglycemia, %	54	33	
Impaired fasting glucose, %	17	25	
Diabetes, %	29	40	
eGFR (mL/min), mean ± SD	67 ± 19	65 ± 19	0.1
Low eGFR ^d^, %	38	42	0.3
Serum uric acid (mg/dL), mean ± SD	4.7 ± 0.9	6.8 ± 1	<0.001
Baseline medications, %:			
Statins	18	26	0.01
Anti-diabetic	10	14	0.08
Anti-hypertensive	27	33	0.1
Reported co-morbidities and risk factors, %:			
Hypertension	35	46	0.01
S/P MI	9	11	0.5
S/P CVA	7	9	0.3
Arthritis	16	23	0.08
Malignancy	14	17	0.2
Family history of IHD	10	12	0.2

* Sex-specific serum uric acid levels.

^‡^ Serum uric acid groups: Low- two lower tertiles of serum uric acid levels. High- upper SUA tertile.

^a^ BMI categories: Normal < 25 kg/m^2^, Overweight, 25–29.9 kg/m^2^, Obese- BMI ≥ 30 kg/m^2^

^b^ High total cholesterol was defined as total cholesterol≥200 mg/dl.

^c^ Glycemic state classification: normoglycemic-fasting glucose<100 mg/dl; Impaired fasting glucose- fasting glucose 100–125 mg/dl; Diabetes- fasting glucose ≥126 mg/dl.

^d^ Low eGFR- defined as eGFR <60 (mL/min) according to the Cockcroft and Gault formula.

BMI, body mass index; eGFR, Estimated glomerular filtration rate; MI, Myocardial infraction; CVA, Cerebrovascular accident; IHD, Ischemic heart disease.

Mean age at baseline was 72 ± 7 years and 416 (49%) were females. 612 (71.9%) were married and only 106 (12.5%) participants had academic education. The cohort was relatively healthy at baseline with 90 (11%) current smokers, 325 (38%) had hypertension, 83 (10%) had previous MI, 93 (28%) had family history of IHD and 130 (16%) had a history of cancer. Mean systolic blood pressure was 137 ± 20 mmHg, Mean BMI was 28 ± 6.2 kg/m^2^ and 243 (30%) were classified as obese (BMI≥30 kg/m^2^). 154 (18%) individuals reported having non-Gout arthritis, while only 4 (0.5%) individuals reported consuming low protein diet and 14 (1.6%) were vegans/vegetarians. Participants were mainly treated with anti-hypertensive medications 244 (46%), statins 173 (33%), and anti-diabetic medications 95 (18%).

Compared with individuals without diuretics treatment (n = 856), those treated with diuretics and excluded from the final cohort (n = 107), were less physically active (35.5% vs. 52.0%, p = 0.001), had higher prevalence of baseline hypertension (77.4% vs. 38.3%, p<0.001), CVA (15.0% vs. 7.4%, p<0.001), MI (21.5% vs. 9.8%, p<0.001), higher mean creatinine levels (1.2 mg/dl±0.8 vs. 1.0 mg/dl±0.2, p<0.001), higher SUA (6.1 ±1.4 mg/dl vs. 5.3 ±1.8 mg/dl, p<0.001), higher BMI (29.4 ±4.6 kg/m^2^ vs. 28.3 ±6.2 kg/m^2^, p = 0.03), older mean age (75 ±7 years vs. 71.9 ±7 years, p<0.001) and higher systolic blood pressure (143±22 mmHg vs. 136.9±19 mmHg, p = 0.01).

Compared to individuals with low SUA (n = 584), participants with high SUA (n = 267) were significantly more obese (23% vs. 41%, p < .001) with higher prevalence of diabetes (29% vs. (40%), p<0.001) and baseline hypertension (35% vs. 46%, p = 0.01), higher values of triglycerides (139 mg/dl [IQR-77] vs. 112 mg/dl [IQR-68], p<0.001) and were treated more frequently with statins (26% vs. 18%, p = 0.01).

### ECG findings

ECG abnormalities were observed in 518 (60.9%) participants (S1 Table). The most common abnormality was conduction defect, observed in 262 (30.8%) subjects. Ischemic changes, defined as ischemic damage without MI or old MI, were observed in 85 (10.0%) participants. In the high SUA group, 36 (13.5%) participants presented with Ischemic changes on ECG compared with 49 (8.4%) in the low SUA group (p = 0.02).

Multivariable logistic regression model (Table 2), adjusted for age, sex, ethnicity, current smoking, blood pressure, current physical activity, low eGFR, cholesterol, abnormal fasting glucose, and past myocardial infraction, showed greater adjusted prevalence odds for ischemic changes on ECG with high SUA (OR = 1.8, 95%CI: 1.1–2.9, p = 0.03).

**Table 2:** Logistic regression models for the association between serum uric acid and ischemic changes on ECG.

		Univariate model			Multivariable model		
Characteristic	Reference category	OR	95% CI	p -value	OR	95% CI	p -value
Age	1- year increment	1.0	0.99, 1.0	0.8	1.0	0.98, 1.1	0.5
Sex, Male	Female	1.8	1.0, 3.1	0.04	1.5	0.8, 2.5	0.2
Origin:	Yemen						
Middle East		1.5	0.7, 3.0	0.3	1.5	0.7, 3.2	0.3
North Africa		1.8	0.8, 3.8	0.1	1.4	0.6, 3.2	0.5
Europe-America		1.9	0.9, 3.7	0.06	1.8	0.9, 3.8	0.1
Smoking status, current	Never/past	2.0	1.1, 3.6	0.03	1.8	0.9, 3.6	0.09
Physical activity, current	Never/past	0.8	0.5, 1.3	0.4	0.7	0.6, 0.9	0.001
Systolic blood pressure	10 mm/Hg increment	1.0	0.9, 1.1	0.8	1.0	0.9, 1.2	0.7
Low eGFR ^a^	Normal eGFR	1.6	1.6, 2.6	0.03	1.4	0.8, 2.5	0.3
Abnormal fasting glucose ^b^	Normal fasting glucose	1.5	0.9, 2.3	0.1	1.3	0.8, 2.2	0.3
Obese* ^c^	Non obese	1.3	0.8, 2.1	0.3	-	-	-
Statin use*	No use	0.6	0.3, 1.0	0.1	-	-	-
Total cholesterol	10 mg/dl increment	0.9	0.9, 0.96	0.001	0.95	0.9, 1.0	0.1
Past MI	None	8.3	4.9, 14.0	<0.001	6.8	3.8, 12.0	<0.001
High SUA ^d^	Low SUA	1.7	1.1, 2.7	0.02	1.8	1.1, 2.9	0.03

^a^ Low eGFR- defined as eGFR <60 (mL/min) according to the Cockcroft and Gault formula.

^**b**^ Abnormal fasting glucose-defined as fasting glucose ≥ 100 mg

^**c**^ Obese- BMI ≥ 30 kg/m^2^, Non-Obese- BMI < 30 kg/m^2^

^**d**^ Low SUA- two lower tertiles of serum uric acid levels. High SUA- upper SUA tertile.

* The variable was not included in the multivariable model.

OR–Odds Ratio; CI–Confidence interval; eGFR, Estimated glomerular filtration rate; MI, Myocardial infraction; SUA, serum uric acid.

Similarly, past MI was associated with higher prevalence odds for ischemic changes (OR = 6.8, 95%CI: 3.8–12.0, p<0.001) whereas current physical activity was associated with lower prevalence odds for ischemic changes (OR = 0.7, 95%CI: 0.6–0.9, p<0.001).

**Fig 1:**
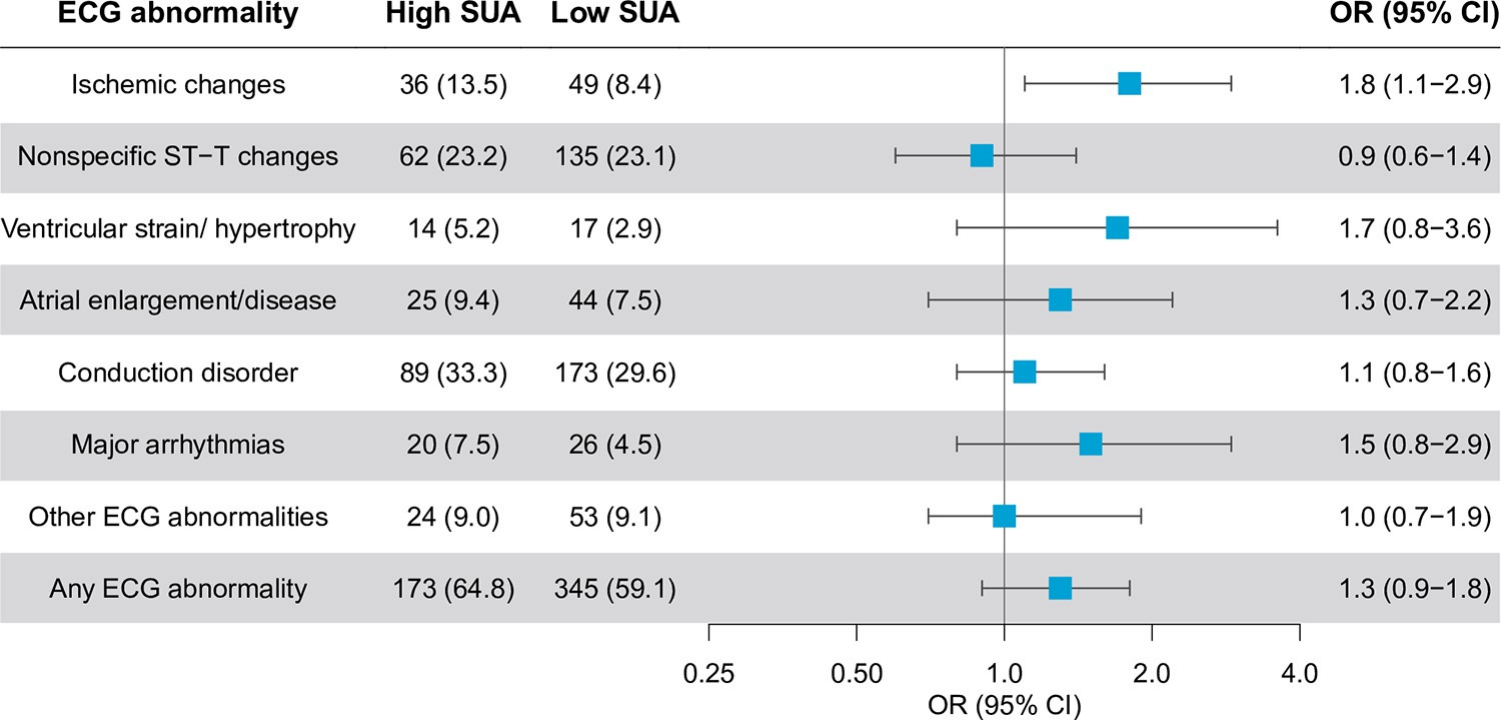
Odds Ratios for the association between upper tertile of sex-specific serum uric acid and ECG abnormalities. Multivariable ^a^ logistic regression analysis. ^a^ Adjusted for age, sex, origin, physical activity, low eGFR, systolic blood pressure, fasting glucose, total cholesterol, current smoking, past MI, high serum uric acid.

No statistically significant associations were observed between SUA and other ECG findings in the adjusted multivariable analyses (Fig 1, S2 Table).

### All-cause mortality

During a median follow up of 14 [IQR-7] years, 380 (44.7%) participants died and 11,029 person years were accrued. Until December 2016, 309 (36.3%) causes of deaths were reported, of them 72 (23.3%) were attributed to cardiovascular disease as the primary cause of death, 70 (22.6%) due to infectious disease, 46 (14.9%) were malignancy associated, 23 (7.4%) were due to respiratory illness, and 95 (30.7%) died from other causes.

**Fig 2:**
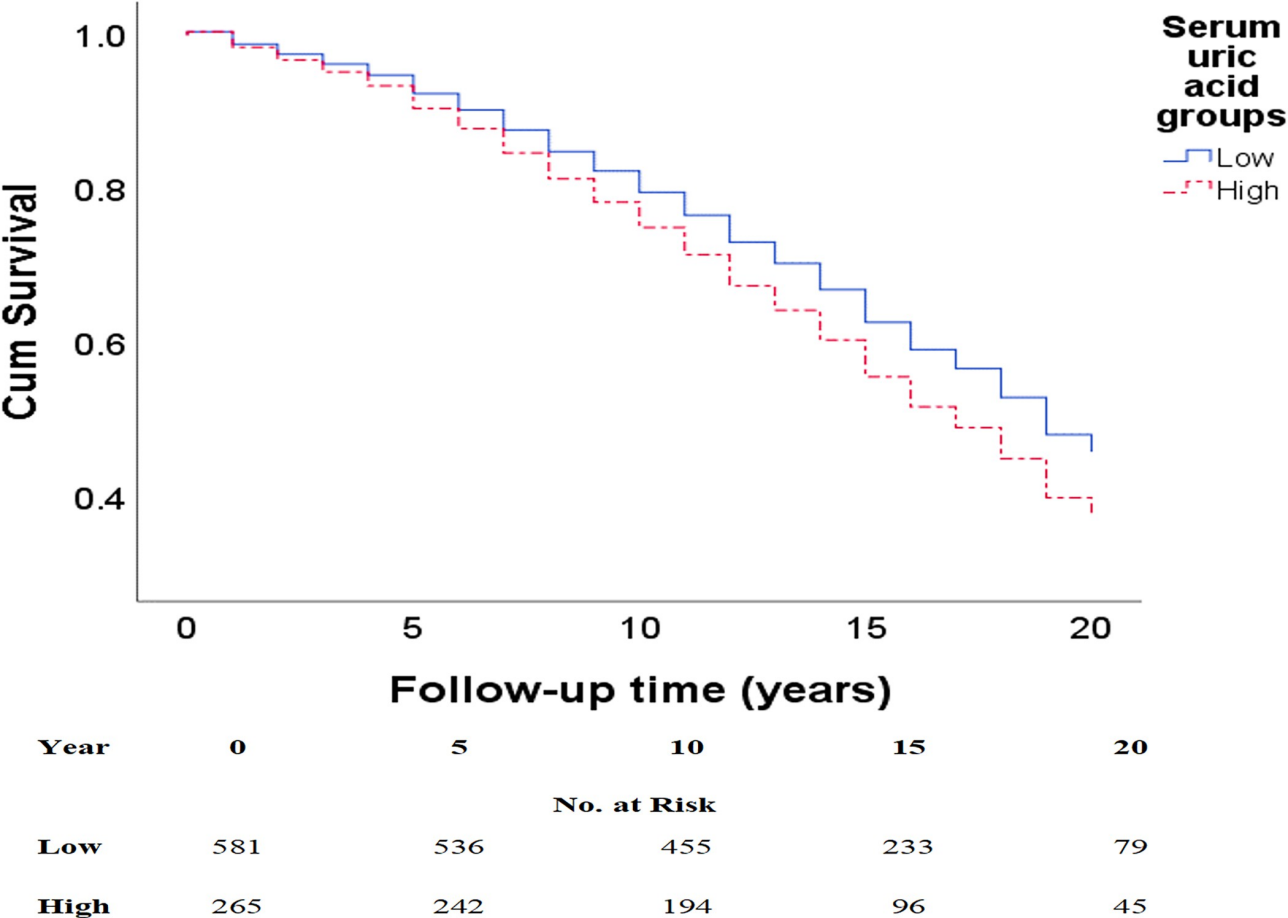
Adjusted ^a^ Cox regression survival curves according to serum uric acid tertiles for all-cause mortality. ^a^ Adjusted for age and sex. Mean survival time for all-cause mortality in the low SUA tertiles (T1-2) was 15.3 (95%CI, 14.8–15.8) years and 14.3 (95%CI, 13.5–14.7) years in the upper SUA tertile (T3), p = 0.03. *Please note that the y-axis doesn’t reach zero.

Survival curves according to Cox regression (Fig 2), adjusted for sex and age, demonstrated a significant shorter times until death for subjects in the high SUA group, p = 0.03.

High SUA was significantly associated with a 30% increased risk for all-cause mortality in the multivariable Cox regression model (Table 3) adjusted for age, sex, ethnicity, smoking status, blood pressure, sports related physical activity level, eGFR, cholesterol, fasting glucose, and past myocardial infraction, (HR = 1.3, 95%CI: 1.0–1.6, p = 0.03) as compared to the lower SUA tertiles.

**Table 3:** Cox regression models for associations between SUA and all-cause mortality.

Characteristic	Reference category	Univariate model			Multivariable model		
		HR	95% CI	p -value	HR	95% CI	p -value
Age	1 year increment	1.1	1.0, 1.1	<0.001	1.1	1.0, 1.1	<0.001
Sex, Male	Female	1.2	1.0, 1.5	0.04	1.2	0.9, 1.5	0.1
Origin:	Yemen						
Middle East		0.9	0.7, 1.3	0.8	0.9	0.7, 1.3	0.8
North Africa		0.9	0.7, 1.1	0.3	0.8	0.6, 1.2	0.3
Europe-America		0.9	0.7, 1.1	0.3	0.8	0.6, 1.2	0.08
Smoking status, current	Never/past	1.0	0.7, 1.4	0.9	1.2	0.8, 1.7	0.4
Physical activity, current	Never/past	0.6	0.5, 0.7	<0.001	0.7	0.6, 0.9	0.001
Systolic blood pressure	10 mm/Hg increment	1.0	1.0, 1.01	<0.001	1.1	1.0, 1.2	0.001
Low eGFR ^a^	Normal eGFR	2.2	1.8, 2.8	<0.001	1.3	1.0, 1.7	0.049
Abnormal fasting glucose ^b^	Normal fasting glucose	1.2	1.0, 1.5	0.04	1.1	0.9, 1.4	0.4
Obese* ^c^	Non obese	1.2	0.9, 1.5	0.1	-	-	-
Statin use*	No use	1.1	0.8, 1.4	0.5	-	-	-
Total cholesterol	10 mg/dl increment	0.99	0.98, 0.99	0.02	1.0	0.9, 1.0	0.2
Past MI	None	1.9	1.4, 2.6	<0.001	1.9	1.4, 2.6	<0.001
High SUA ^d^	Low SUA	1.3	1.1, 1.6	0.01	1.3	1.0, 1.6	0.03

^a^ Low eGFR- defined as eGFR <60 (mL/min) according to the Cockcroft and Gault formula.

^**b**^ Abnormal fasting glucose-defined as fasting glucose ≥ 100 mg

^**c**^ Obese- BMI ≥ 30 kg/m^2^, Non-Obese- BMI < 30 kg/m^2^

^**d**^ Low SUA- two lower tertiles of serum uric acid levels. High SUA- upper SUA tertile.

* The variable was not included in the multivariable model.

HR–Hazard Ratio; CI–Confidence interval; eGFR, Estimated glomerular filtration rate; MI, Myocardial infraction; SUA, serum uric acid.

An expected significant greater risk for mortality was also observed with age, such that for every 1 year increment there was an observed 10% higher death risk (HR = 1.1, 95%CI: 1.0–1.1, p<0.001), systolic blood pressure (HR = 1.1, 95%CI: 1.0–1.2, p = 0.001) for increments of 10 mmHg, low eGFR (HR = 1.3, 95%CI: 1.0–1.7, p = 0.049) and past MI (HR = 1.9, 95%CI: 1.4–2.6, p<0.001), while current physical activity was associated with lower 14 years risk for all-cause mortality (HR = 0.7, 95%CI: 0.6–0.9, p = 0.001).

### Interaction analysis

We did not detect any significant interaction (S3 Table) between SUA tertiles and physical activity (P for interaction = 0.4), eGFR (P for interaction = 0.2), fasting glucose (P for interaction = 0.2), BMI (P for interaction = 0.2), past MI (P for interaction = 0.9), sex (P for interaction = 0.9), age (P for interaction = 0.5) or smoking (P for interaction 0.8) in a multivariate model.

## Discussion

The current study, including 851 community dwelling older men and women, free of known Gout or diuretics use, showed 80% greater prevalence odds for ischemic changes on ECG and a 30% greater risk for 14-year all-cause mortality, among those in the upper sex-specific tertile of SUA compared to the lower tertiles.

### SUA and ECG findings

Although high SUA was previously associated with ECG findings such as atrial fibrillation and LVH, its association with other abnormalities such as ischemic changes is still undetermined. Several large observational studies [24] as well as large systematic reviews with meta-analysis [10] established a significant and increased risk for atrial fibrillation among individuals with high SUA. Similar findings were also observed with ventricular hypertrophy and tachyarrhythmia’s on 1,557 individuals, as part of the Brisighella Heart Study, and after only 4-years of follow-up [11]. Furthermore, the study [11] also found an increased risk for past myocardial infarction (Q wave abnormality) among individuals with high SUA. Correspondingly, a cross sectional study of 5,880 North American adults, without known CV disease at baseline, from the National Health and Nutrition Examination Survey (NHANES) III [25], found a 15% greater risk for a subclinical myocardial injury (defined by ECG based Cardiac Infarction Injury Score (CIIS) ≥10 units [26], with high SUA, although, the association was significant among women only. Our findings are in congruence with these publications, showing an 80% greater odds for ischemic changes (defined as ischemic damage without MI or old MI) on ECG among individuals in the upper tertile of the sex-specific SUA. Our findings further underscore the association of SUA with CV morbidity, exemplified by incidental ischemic changes on ECG recorded in the framework of a prospective cohort study, without awareness of overt myocardial ischemia, calling for further investigation.

### SUA and all-cause and CV mortality

The role of SUA in the pathogenesis of CV morbidity and mortality remains controversial.

Although current data support the association between SUA and CVD, it is still questionable whether the effect is causal or that SUA serves as a mediator for other known CV risk factors [27–29]. Several mechanisms were previously postulated in order to offer a reasonable explanation for its casual role, such as inflammatory response, renal microcirculation vascular changes and renin-angiotensin system activation [5, 30], endothelial dysfunction, oxidative stress and subclinical atherosclerosis [31]. Moreover, SUA was previously associated with several metabolic diseases such as non-alcoholic fatty liver [32] type 2 diabetes mellitus [33], and hypertension [34]. In a systematic review, Zhao et al. [16] demonstrated using meta-analysis of eleven studies with 172,123 participants, that elevated SUA was associated with increased risk for all-cause mortality (RR = 1.24, 95%CI: 1.09–1.42), an association that was modified by sex, exemplified by an increased risk for mortality among men (RR = 1.23, 95%CI: 1.08–1.42) but not among women (RR = 1.05, 95%CI: 0.79–1.39). Similar findings were also observed as part of another robust systematic review with meta-analysis, comprised of 341,389 adults, showing higher risk for all-cause and CV mortality with hyperuricemia [35].

In older participants, Hu et al. [36] observed that among 1,093 Taiwanese participants aged 65 and older, with a 10 year follow-up, increased risk for both cardiovascular and all-cause mortality were noted in the upper quartile of SUA compared with the lowest quartile (adjusted HR = 3.4, 95%CI: 1.2–9.9, and HR = 1.82, 95%CI: 1.02–3.26 respectively). Correspondingly, similar findings were observed with all-cause mortality among 3,926 Polish adults aged 65 years or above, not treated with xanthine oxidase inhibitors, albeit this effect was only significant among men in the multivariable model [37].

Other studies failed to show a significant association between SUA and all-cause mortality in older adults in the total population and among type 2 diabetes patients [18, 38, 39]. However, these studies were of relatively short duration, incomplete adjustment for confounders or did not exclude participants with Gout or diuretics treatment.

In respect to the current data, our findings suggest that higher SUA is associated with a greater all-cause mortality risk among community dwelling older men and women not treated with diuretics and without Gout at baseline. This association holds after adjusting for other known CV risk factors and was not modified by renal function, fasting glucose, BMI, past MI, physical activity, smoking or sex, as demonstrated by the interaction analysis. These findings further reinforce a potential independent association between SUA and earlier mortality, although additional prospective studies are needed. This association should be further examined in order to establish SUA role in CV morbidity and mortality.

### SUA cut-offs for all-cause mortality

Currently, a consensus on SUA optimal values is still lacking in the general population and specifically among older adults. Previously, SUA levels of >7 mg/dl in men and > 6 mg/dl in women were considered as abnormal [40, 41], however accumulating data showed an increased risk for all-cause mortality with lower values as well [14].

Kuo et al. [14] showed a U-shaped association between SUA and all-cause mortality on 354,110 relatively young adults, with a mean follow-up of 4.6 ± 2.6 years, which was attenuated in individuals with CV risk factors. The risk for all-cause mortality was significantly more pronounced among participants in the low strata of SUA (≤2.9 mg/dl) and in the higher strata (≥11mg/dl) (HR = 1.76, 95%CI: 1.57–1.98 and HR = 1.75, 95%CI: 1.62–1.9 respectively) as compared to SUA of 5–6.9 mg/dl.

In a recently published study of 19,769 Israeli healthy middle-aged adults [42], SUA values of ≥ 5.5 mg/dl in men and ≥3.7 mg/dl in women were associated with increased risk for CV morbidity as well as non-cancer mortality over a median follow-up period of 6 years.

In the elderly population, Tseng et al. [43] found that among Taiwanese older adults, aged 65 years and older and with a median follow‐up of 5.8 years, higher risk for all‐cause mortality and CV mortality were observed with SUA values of ≥8 mg/dl and ≥7 mg/dl in men and women respectively. Individuals with SUA ≥10 mg/dl had the highest risk for all‐cause and CVD mortality while SUA values of < 4 mg/dl were also associated with increased mortality in malnourished adults. Importantly, SUA levels of 4 to <8 mg/dl were associated with the lowest rates of all‐cause and CV mortality. Winder et al. [37] found that among Polish older adults, aged ≥65 years, increased risk for mortality was noted in the crude model with SUA ≥ 7 mg/dl, although after adjustment, the association was significant in men alone with SUA ≥ 8 mg/dl. Importantly, Dutta et al. [44] showed that among individuals aged 70 and older, SUA ≥ 7 mg/dl was associated with increased CVD mortality while the addition of SUA to the Framingham Cardiovascular Risk Score resulted in improved prediction of mortality in older adults.

Compared with previous studies, our findings support even lower thresholds for long-term all-cause mortality among older adults, demonstrated by a significant risk for mortality with SUA ≥5.3 mg/dl for women and ≥ 6.2 mg/dl for men. Moreover, our results suggest that optimal SUA levels should be determined according to age in addition to sex.

### Limitations

Several limitations of this study should be acknowledged. First, this study included individuals who survived to reach the 3^rd^ phase of the GOH longitudinal study. It is therefore comprised of the younger and the healthier participants of the cohort. Nevertheless, this does not reduce the relevancy of our findings to elderly men and women living in the community. Second, due to the relatively low rates of cardiovascular mortality, we were under-powered to examine the association between SUA and CV mortality. Nonetheless, since CV mortality is the major cause of death in our cohort, it was most likely the main driver of the observed association with mortality.

Despite these limitations, the current analysis presents significant advantages, such as equal representation of both men and women in an ethnically diverse sample of the Israeli- Jewish population; adjustments of SUA according to sex; relatively long follow-up period; extensive data on socioeconomic characteristics, baseline pharmacologic treatment, co-morbidities and specific diets addressing these morbidities and risk factors, and the exclusion of individuals treated with diuretics or with the diagnosis of Gout.

## Conclusions

Non-Gout sex-specific hyperuricemia was independently associated with ischemic changes on ECG and with long-term all-cause mortality among older adults. Furthermore, our findings support lower than previously suggested SUA thresholds to be associated with all-cause mortality. While further research is needed to explore the biological mechanisms underlying these findings, and to establish the cut-off values for hyperuricemia, SUA may be considered as an independent biomarker for CV morbidity and mortality in the elderly population.

## Supporting information

S1 Table Classification and prevalence of ECG findings. (DOCX)

S2 Table Unadjusted and adjusted^a^ logistic regression models for ECG findings. ^a^ Adjusted for: age, sex, origin, obesity, systolic blood pressure, high cholesterol, smoking, statin use, baseline MI, low eGFR, physically active and abnormal fasting glucose. ^‡^ Serum uric acid groups: Low- two lower tertiles of serum uric acid levels. High- upper SUA tertile. *Odds ratio.
(DOCX)

S3 Table Multivariable Cox regression model for all-cause mortality; interaction analysis between SUA (upper sex specific tertile vs. lower tertiles) and cohort’s characteristics. ^‡^ P for interaction between variable and serum uric acid in multivariable model. Adjusted for age, sex, origin, physical activity, low eGFR, systolic blood pressure, fasting glucose, total cholesterol, current smoking, past MI, high serum uric acid. ^a^ Low eGFR- defined as eGFR <60 (mL/min) according to the Cockcroft and Gault formula.; ^**b**^ Fasting glucose group, abnormal- fasting glucose ≥ 100 mg; ^**c**^ BMI groups, Obese- BMI ≥ 30 kg/m^2^, non -obese- BMI<30 kg/m^2^. ^**d**^ Age groups, <65 years, ≥65 years. eGFR, Estimated glomerular filtration rate; MI, Myocardial infraction; SUA, serum uric acid. BMI, body mass index.
(DOCX)
