# Supplementary material for: Sex specific serum uric acid levels are associated with ischemic changes on ECG and with 20-year all-cause mortality among older adults
Source: PLoS One. 2023 Mar 30;18(3):e0283839. doi: 10.1371/journal.pone.0283839 (PMC10062641; doi:10.1371/journal.pone.0283839)
Supplement: S1 Table — (DOCX) [file pone.0283839.s001.docx]

**S1 Table. Classification and prevalence of ECG findings**

| **N (%)** | **Description** | **ECG category** |
| --- | --- | --- |
| 518 (61.0) |  | Any ECG abnormality |
| 262 (30.6) | Including complete left bundle branch block | Conduction disorder |
| 197 (23.2) |  | Nonspecific ST-T changes |
| 85 (10.0) | Ischemic damage without MI or old MI | Ischemic changes |
| 69 (8.1) |  | Atrial enlargement/disease |
| 46 (5.4) |  | Major arrhythmias |
| 31 (3.6) |  | Ventricular strain/ hypertrophy |
| 77 (9.0) |  | Other |
