# Supplementary material for: Sex specific serum uric acid levels are associated with ischemic changes on ECG and with 20-year all-cause mortality among older adults
Source: PLoS One. 2023 Mar 30;18(3):e0283839. doi: 10.1371/journal.pone.0283839 (PMC10062641; doi:10.1371/journal.pone.0283839)
Supplement: S2 Table — a Adjusted for: age, sex, origin, obesity, systolic blood pressure, high cholesterol, smoking, statin use, baseline MI, low eGFR, physically active and abnormal fasting glucose. ‡ Serum uric acid groups: Low- two lower tertiles of serum uric acid levels. High- upper SUA tertile. *Odds ratio. (DOCX) [file pone.0283839.s002.docx]

**S2 Table. Unadjusted and adjusted**^a^ **logistic regression models for ECG findings**

| **Other**  n**=**77 | **Major arrhythmias**  n**=**46 | **Conduction disorder**  n**=**262 | **Atrial enlargement/disease**  n**=**69 | **Ventricular strain/ hypertrophy**  n=31 | **Nonspecific ST-T changes**  n=197 | **Any ECG abnormality**  n=518 | **SUA**^‡^ **(highest vs lower tertiles)** |
| --- | --- | --- | --- | --- | --- | --- | --- |
| 0.99 (0.6-1.6) | 1.7 (0.9-3.2) | 1.2 (0.9-1.6) | 1.3 (0.8-2.1) | 1.8 (0.9-3.8) | 1.0 (0.7-1.4) | 1.3 (0.9-1.7) | **Unadjusted OR*** |
| 0.9 | 0.07 | 0.3 | 0.4 | 0.1 | 0.9 | 0.1 | **P-value** |
| 1.0 (0.7-1.9) | 1.5 (0.8-2.9) | 1.1 (0.8-1.6) | 1.3 (0.7-2.2) | 1.7 (0.8-3.6) | 0.9 (0.6-1.4) | 1.3 (0.9-1.8) | **Adjusted OR*** |
| 0.7 | 0.2 | 0.5 | 0.4 | 0.2 | 0.8 | 0.1 | **P-value** |

^a^ Adjusted for: age, sex, origin, obesity, systolic blood pressure, high cholesterol, smoking, statin use, baseline MI, low eGFR, physically active and abnormal fasting glucose.

^‡^ Serum uric acid groups: Low- two lower tertiles of serum uric acid levels. High- upper SUA tertile.

*Odds ratio.
