# Supplementary material for: Sex specific serum uric acid levels are associated with ischemic changes on ECG and with 20-year all-cause mortality among older adults
Source: PLoS One. 2023 Mar 30;18(3):e0283839. doi: 10.1371/journal.pone.0283839 (PMC10062641; doi:10.1371/journal.pone.0283839)
Supplement: S3 Table — ‡ P for interaction between variable and serum uric acid in multivariable model. Adjusted for age, sex, origin, physical activity, low eGFR, systolic blood pressure, fasting glucose, total cholesterol, current smoking, past MI, high serum uric acid. a Low eGFR- defined as eGFR <60 (mL/min) according to the Cockcroft and Gault formula.; b Fasting glucose group, abnormal- fasting glucose ≥ 100 mg; c BMI groups, Obese- BMI ≥ 30 kg/m2, non -obese- BMI<30 kg/m2. d Age groups, <65 years, ≥65 years. eGFR, Estimated glomerular filtration rate; MI, Myocardial infraction; SUA, serum uric acid. BMI, body mass index. (DOCX) [file pone.0283839.s003.docx]

Table S3. Multivariable Cox regression model for all-cause mortality; interaction analysis between SUA (upper sex specific tertile vs. lower tertiles) and cohort's characteristics.

| Characteristic | Category | Mortality  n (%) | P for interaction^‡^ | 95% CI |
| --- | --- | --- | --- | --- |
| Physical activity | Current | 166 (43.7) | 0.4 | 0.5, 1.3 |
|  | Never/past | 214 (56.3) |  |  |
| Low GFR ^a^ | Low | 192 (51.9) | 0.8 | 0.6, 1.6 |
|  | Normal | 178 (48.1) |  |  |
| Fasting glucose groups ^b^ | Abnormal | 229 (61.7) | 0.2 | 0.8, 2.0 |
|  | Normal | 142 (38.3) |  |  |
| BMI groups ^c^ | Obese | 121 (33.2) | 0.2 | 0.8, 2.0 |
|  | Non-obese | 243 (66.8) |  |  |
| S/P MI | Yes | 53 (14) | 0.4 | 0.7, 2.4 |
|  | No | 326 (86) |  |  |
| Sex | Male | 209 (55) | 0.9 | 0.7, 1.5 |
|  | Female | 171 (45) |  |  |
| Age ^d^ | <65 | 24 (6.3) | 0.5 | 0.6, 3.0 |
|  | ≥65 | 356 (93.7) |  |  |
| Smoking | Current | 41 (10.8) | 0.8 | 0.5, 1.6 |
|  | Never/past | 339 (89.2) |  |  |

^‡^ P for interaction between variable and serum uric acid in multivariable model. Adjusted for age, sex, origin, physical activity, low eGFR, systolic blood pressure, fasting glucose, total cholesterol, current smoking, past MI, high serum uric acid.

^a^ Low eGFR- defined as eGFR <60 (mL/min) according to the Cockcroft and Gault formula.; **^b^** Fasting glucose group, abnormal- fasting glucose ≥ 100 mg; **^c^** BMI groups, Obese- BMI ≥ 30 kg/m^2^, non -obese- BMI<30 kg/m^2^. **^d^** Age groups, <65 years, ≥65 years.

eGFR, Estimated glomerular filtration rate; MI, Myocardial infraction; SUA, serum uric acid. BMI**,** body mass index**.**
